# New Appliances and Things Medical

**Published:** 1904-10-15

**Authors:** 


### NEW APPLIANCES AND THINGS MEDICAL.

### THE ALLENBURYS MILK AND CEREAL DIET PANCREATISED.

(Allen and Hanburys, Limited, London.)

The food before us is made from pure milk containing a high percentage of cream, and whole wheat. Both these substances, however, are, during the process of manufacture, partially digested. This food is identical with the food which was introduced by these manufacturers some little time ago under the name "The Allenburys Food No. 4." The food is recommended in all conditions in which an emphatically unirritating diet is indicated, and is a perfect food in so far as it contains all the elements of a typical diet in adequate proportion and in an easily digestible and assimilable form. When made either with milk or water a pleasant beverage results, which is free from the sweet taste so often present in similar preparations. We think the food is to be recommended, and should have a wide sphere of usefulness.

### Liberal Views and the Medical Profession.

Speaking at the annual dinner of the South-West London Medical Society, Dr. White, the president, remarked that it was their custom to invite members of other medical societies to read papers at their meetings. The practice may well be commended as worthy of imitation. The past year has been one of international congresses, and even now we have a large and representative gathering of French surgeons and physicians in London. It is evident, therefore, that the value of interchange of ideas among the nations is properly valued; and it may be hoped that the same liberal spirit will soon pervade the smaller spheres of medical thought represented by the metropolitan and provincial medical societies, many of which may be regarded as too exclusive, and especially so in the case of those which are interested in one particular "speciality." If more liberal habits were cultivated in this respect the recently renewed arguments in favour of a single comprehensive "Royal Society of Medicine" would lose much of their force.

